# Soil Drenching with Wood Distillate Modifies the Nutritional Properties of Chickpea (*Cicer arietinum* L.) Seeds by Increasing the Protein Content and Inducing Targeted Changes in the Proteomic Profile

**DOI:** 10.3390/plants14132046

**Published:** 2025-07-03

**Authors:** Rossana De Salvo, Riccardo Fedeli, Alfonso Carleo, Luca Bini, Stefano Loppi, Laura Bianchi

**Affiliations:** 1 Laboratory of Functional Proteomics, Department of Life Sciences, Siena University, 53100 Siena, Italy; rossana.desalvo@student.unisi.it (R.D.S.); luca.bini@unisi.it (L.B.); laura.bianchi@unisi.it (L.B.); 2 BioAgry Lab, Department of Life Sciences, University of Siena, 53100 Siena, Italy; loppi@unisi.it; 3 Laboratory of Molecular Medicine and Genomics, Department of Medicine, Surgery and Dentistry “Scuola Medica Salernitana”, University of Salerno, 84081 Baronissi, Italy; alcarleo@unisa.it; 4 NBFC—National Biodiversity Future Center, 90121 Palermo, Italy

**Keywords:** differential chickpea proteomics, micro- and macronutrient profiles, naturally fortified food, legume nutritional value, plant productivity, plant based protein transition, pyroligneous acid

## Abstract

The production of food with a naturally enriched protein content is a strategic response to the growing global demand for sustainable protein sources. Wood distillate (WD), a by-product of the pyrolysis of woody biomass, has previously been shown to increase the protein concentration and bioavailability in chickpea seeds. Here, we evaluated the effect of 0.5% (*v/v*) WD soil drenching on chickpea productivity, nutritional profile, and proteomic pattern. WD treatment significantly improved the yield by increasing plant biomass (+144%), number of pods and seeds (+148% and +147%), and seed size (diameter: +6%; weight: +25%). Nutritional analyses revealed elevated levels of soluble proteins (+15%), starch (+11%), fructose (+135%), and polyphenols (+14%) and a greater antioxidant capacity (25%), alongside a reduction in glucose content, albeit not statistically significant, suggesting an unchanged or even lowered glycemic index. Although their concentration decreased, Ca (−31%), K (−12%), P (−5%), and Zn (−14%) in WD-treated plants remained within normal ranges. To preliminary assess the quality and safety of the protein enrichment, a differential proteomic analysis was performed on coarse flours from individual seeds. Despite the higher protein content, the overall protein profiles of the WD-treated seeds showed limited variation, with only a few storage proteins, identified as legumin and vicilin-like isoforms, being differentially abundant. These findings indicate a general protein concentration increase without a major alteration in the proteoform composition or differential protein synthesis. Overall, WD emerged as a promising and sustainable biostimulant for chickpea cultivation, capable of enhancing both yield and nutritional value, while maintaining the proteomic integrity and, bona fide, food safety.

## 1. Introduction

Chickpea (*Cicer arietinum* L.) is a widely grown legume, valued for its rich nutritional profile, particularly its high protein content, which plays a relevant role in supporting human nutrition and health [1]. In the context of a rapidly growing global population, the demand for dietary proteins is escalating, prompting a search for sustainable and efficient protein sources [2]. As a valuable plant-based option, chickpeas are a promising component of more sustainable dietary patterns, contributing to the diversification of protein intake and helping to reduce the reliance on animal-derived products [3]. The shift towards plant-based diets is driven by multiple factors, including the need to mitigate the environmental impact of intensive livestock farming. Conventional animal agriculture is associated with substantial resource consumption, including land, water, and feed, as well as significant greenhouse gas emissions, which contribute greatly to climate change [4,5]. Moreover, the ethical considerations of animal welfare and the risks of zoonotic diseases further underscore the urgency of finding alternative protein sources. In this context, legumes, whose cultivation requires far fewer natural resources compared to animal farming, provide an opportunity for promoting a more sustainable and resource-efficient food system.

In addition to their nutritional benefits, chickpeas support ecological agricultural practices by improving the soil health through nitrogen fixation. This process reduces the need for synthetic fertilizers and minimizes the environmental impact compared to that of conventional farming methods [6]. The promotion of chickpeas as a staple in plant-based diets aligns with broader goals of reducing ecological footprints and promoting food security [7,8].

Furthermore, traditional agricultural practices frequently rely on synthetic pesticides and fertilizers, contributing to soil degradation, water pollution, and negative impacts on biodiversity [9,10,11]. These environmental concerns have prompted the search for sustainable bio-based alternatives. Among them, wood distillate (WD), a liquid by-product of the pyrolysis of woody biomass, has gained attention for its potential to enhance crop growth and nutritional quality [12,13]. Also known as wood vinegar or pyroligneous acid, WD is produced from the thermal decomposition of wood in the absence of oxygen [12]. The process yields a complex mixture of over 300 organic compounds, including acetic acid, methanol, and various phenols and aldehydes [14,15].

WD is currently largely investigated for its biostimulant properties in agriculture, with foliar spraying and soil drenching being the main delivery methods [16,17]. The former involves applying the WD directly to the leaves, where it is absorbed through the foliage and delivers nutrients and bioactive compounds, supporting plant health and improving the resistance to pests [12,18]. Alternatively, soil drenching involves the use of WD in the soil, where it may improve the soil fertility, support microbial activity, and promote the overall plant growth by primarily enhancing the root system growth and development [19,20]. A growing body of evidence indicates that WD can enhance both the yield and quality of various crop species [18,21,22]. However, to our knowledge, no studies have investigated the effects of soil drenching with WD on the yield and nutritional properties of chickpea seeds, particularly with a focus on their proteome profile.

In the current study, we aimed to assess whether soil drenching with WD could influence plant yield parameters and the nutritional properties of chickpea seeds. We also evaluated whether the changes were associated with alterations in the proteome that could raise concerns regarding the nutritional quality and safety of chickpeas.

## 2. Results

### 2.1. Yield Parameters

Soil drenching with 0.5% (*v/v*) WD significantly increased the plant biomass (+144%), number of pods (+148%), number of seeds (+147%), seed diameter (+6%), and seed weight (+25%); differences did not emerge for the plant height (Table 1).

### 2.2. Flour Nutritional Parameters

WD applications significantly reduced the content of Ca (−31%), K (−12%), P (−5%), and Zn (−14%) in the seeds (Table 2).

**Figure 1:**
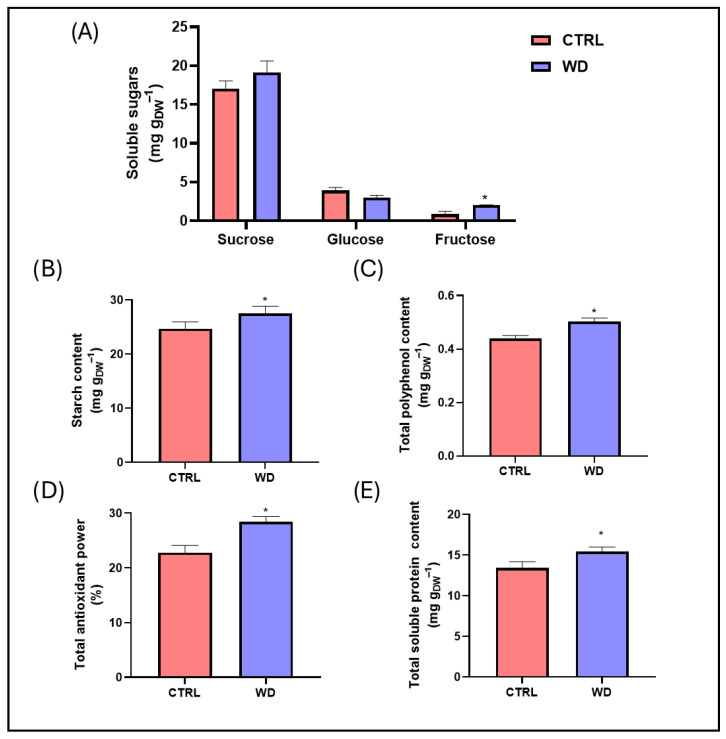
Biochemical parameters (mean ± standard error) of chickpea plants treated (WD) or not treated (CTRL) with 0.5% (*v/v*) wood distillate. * = statistically significant (*p* < 0.05) difference. (**A**) soluble sugars, (**B**) starch, (**C**) total polyphenols, (**D**) total antioxidant power, (**E**) total soluble proteins.

The results of a biochemical analysis (Figure 1) showed that the application of WD significantly increased the content of fructose (+135%) and starch (+11%), the total polyphenols (+14%), the total antioxidant power (+25%), and the total soluble proteins (+15%).

### 2.3. WD-Induced Proteomic Profile Modulation

**Figure 2:**
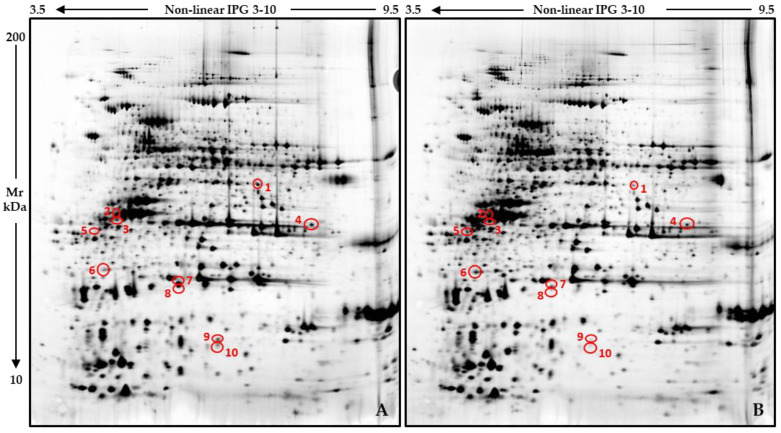
Reference protein patterns of *C. arietinum* seeds from control (**A**) and WD-treated plants (**B**). Red circles and numbers point out differentially abundant protein spots detected by comparing chickpeas from two groups.

In order to evaluate if and how the soil drenching of *C. arietinum* plants with WD may have affected the protein profiles of the produced seeds, a differential proteomics analysis was run on coarse-flour samples individually prepared from single dehydrated chickpeas. The 12 analyzed gels, 6 per condition, are shown in Supplementary Figure S1. According to image analysis, only 10 protein spots were detected to significantly (*p* ≤ 0.05; FC ≥ 2) differ between the two analytical groups (Figure 2).

**Figure 3:**
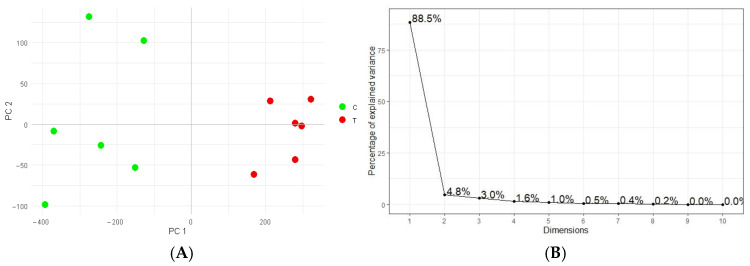
Unsupervised principal component analysis (PCA) of the 10 relevant protein differences (*p* ≤ 0.05 and FC ≥ 2) detected between the 12 analyzed seed samples: 6 controls, green dots, and 6 seeds from WD-treated plants, red dots (**A**). The axonometry combines the first two PCs, PC1/PC2, which described 88.5% and 4.8% of the variance, respectively, as highlighted by the scree plot (**B**).

The results of a PCA (Figure 3) based on the 10 differentially abundant spots revealed a tighter clustering of the WD-treated samples compared to the controls (Figure 3A). The wider dispersion observed among the control seeds may reflect greater heterogeneity in protein expression under non-stress conditions, while the WD treatment may induce a more uniform proteomic response, thus reducing the biological variability. The first two principal components, i.e., PC1 and PC2, accounted for 88.5% and 4.8% of the total variance, respectively, and together sufficiently explained the overall variance in the difference dataset, as confirmed by the scree plot (Figure 3B).

**Figure 4:**
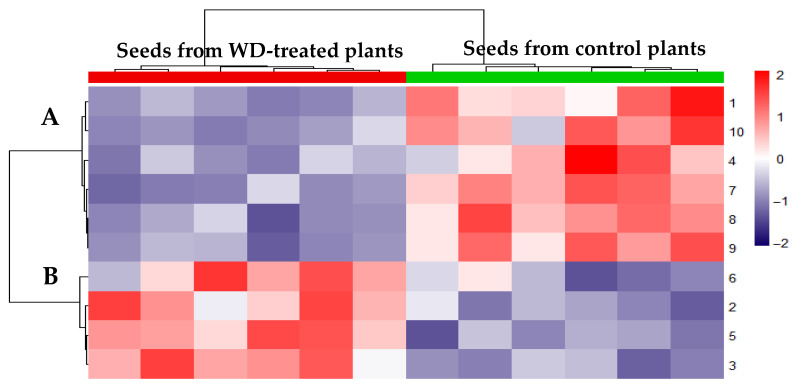
Heatmap of not-scaled Euclidean distances of the abundance values from the detected protein differences. Each column corresponds to an individual seed sample, while each row represents a significant protein difference, as numbered in Figure 3 and listed on the right of the expression matrix. The colour scale (on the right) indicates relative abundance levels, ranging from brilliant red (high abundance) to dark blue (low abundance). Unsupervised clustering grouped the proteins down-regulated by WD in cluster **A**, while the up-regulated ones were included in cluster **B**, as shown by the vertical dendrogram (on the left). This dendrogram groups the 10 differentially abundant protein spots based on similarity of their relative abundance patterns across the samples. The horizontal dendrogram (at the top) clusters the seed samples into two distinct clusters corresponding to WD-treated (red bars) and control plants (green bars), reflecting consistent differences in the protein expression profiles between the investigated conditions.

The relative abundances of the ten differences clustered according to their similarity, as evidenced by the vertical dendrogram in the heatmap (Figure 4). Here, the down-regulated proteins in the seeds from the treated plants were grouped in the vertical cluster A, while the up-regulated ones were included in the vertical cluster B. Although the abundance of each protein difference slightly varied within the same sample set, probably related to the individual heterogeneity of seeds, the heatmap also highlighted a distinct clustering of all the samples from exposed plants in the “seeds from WD-treated plants” group and all the controls in the “seeds from control plants” group (Figure 4, horizontal dendrogram: red and green bars, respectively). Therefore, the abundance profile of the ten detected protein differences clearly distinguished the two types of chickpeas analyzed.

In order to evaluate the biological implications of proteins being deregulated by the WD drenching treatment, MS was applied, and five differences were identified. Interestingly, all of them were plant storage proteins (Table 3).

## 3. Discussion

Soil drenching with WD at 0.5% (*v*/*v*) significantly increased the plant biomass, the number of pods, and the seed number, diameter, and weight, suggesting a strong positive influence on the overall plant productivity. Similar results have been reported for various crop species, including chickpea plants, where WD was applied as a foliar spray at a concentration of 0.25% (*v*/*v*) [23,24]. This effect was likely related to the ability of WD to enhance a plant nutrient uptake and to influence its hormonal pathways, particularly those involving auxins and cytokinins. In fact, recent studies have suggested that WD may act as a beneficial stressor [23]. Its application is supposed to induce a mild stress response in plants, triggering the activation of antioxidant defence mechanisms. Additional studies have shown that WD also influences the content of chlorophyll, a crucial molecule for plant growth and development [25]. This is especially relevant considering that chlorophyll plays a fundamental role in photosynthesis, thereby sustaining plant growth and biomass production [26,27].

The absence of any significant change in the plant height suggests that the WD effects are more specifically directed toward reproductive and yield-related traits. This selective influence may be particularly useful for crop optimization, as it enhances productivity without promoting excessive vegetative growth. In several crops, like chickpeas, maximizing the yield is more economically important than increasing the plant height, often a proxy for vegetative biomass, and shorter plants can also be less susceptible to wind damage and lodging, with considerable agronomic benefits [28]. This lack of height variation could also reflect species-specific growth patterns, as chickpea plants might respond to biostimulants, like WD, differently compared to other crops. Additionally, both the concentration (0.5% - *v/v*) and the specific timing of application may have contributed to the absence of elongation, potentially shifting the physiological balance toward enhanced reproductive development rather than vegetative growth. Such a trade-off between structural and metabolic investment is plausible and warrants further investigation. Additional studies assessing a range of WD doses and application strategies would be valuable to better understand these dynamics.

The statistically significant reduction in the content of essential minerals, namely Ca, K, P, and Zn, in the chickpea flour following WD treatment is a potential downside, given the crucial role these elements play in both plant physiology and human nutrition [29,30]. Nonetheless, the measured levels of the affected minerals remained within the normal ranges reported for chickpea flour [31,32,33], indicating that the observed fluctuations do not imply mineral deficiencies or imbalances that would compromise the seed nutritional quality.

The observed reductions may have been due to competitive uptake dynamics or modifications in ion transport processes induced by WD.

From a biochemical perspective, the improvements observed in the chickpea flour following WD application were notable. Greater antioxidant power and enhanced levels of fructose, sucrose, starch, total polyphenols, and, as outlined above, soluble proteins collectively increased the nutritional and functional value of the chickpeas and chickpea flour. In particular, a rise in the sucrose, fructose, and starch contents may boost the caloric value of chickpea-based foods, with beneficial effects in contexts requiring energy-dense nutrition [34]. Moreover, thanks to the lower glycemic index (GI) of these carbohydrates compared to glucose [35,36] and considering the significant reduction in the glucose content observed in the seeds from the WD-treated plants, the overall glycemic impact of chickpeas following the treatment may remain moderate or even favourable, despite the increase in the total sugar content. Finally, although processing and cooking may modulate the glycemic response to this legume [36], chickpea starch itself is characterized by a relatively low glycemic index, primarily due to its relative amylose content and to the presence of dietary fiber, proteins, and polyphenols, which collectively slow down its digestion and glucose absorption [36,37,38].

Since the accumulation of sugars and starch after WD application has been consistently reported across different plant species [21,39,40], WD is assumed to modulate carbohydrate metabolism pathways and promote the biosynthesis and storage of energy-rich compounds. In our study, WD treatment led to a significant increase in fructose levels, whereas the sucrose and glucose levels showed only small fluctuations that were not statistically significant. Such limited variations, despite previous reports highlighting broader sugar changes, may have been due to differences in the chemical composition of the WD used, which can vary depending on the feedstock and pyrolysis conditions. In addition, the species-specific or varietal characteristics of chickpeas, as well as environmental factors and the timing of sample collection, may have influenced sugar metabolism and partitioning. Under our experimental conditions, fructose accumulation may represent a more sensitive or earlier metabolic response, occurring prior to measurable variations in other soluble sugars.

The increase in the total polyphenols and antioxidant activity is especially promising, as these compounds confer multiple health benefits, including anti-inflammatory and cytoprotective effects, which may help prevent or ameliorate chronic conditions (e.g., cardiovascular disease, diabetes, and cancer) [41,42]. Moreover, WD itself is rich in polyphenols and antioxidants, and its application may stimulate the accumulation of such bioactive molecules in various plant tissues [14]. As legumes are naturally rich in these compounds [43,44,45], further enhancing their polyphenol levels through WD treatment could make chickpeas even more attractive as a functional food. In fact, higher polyphenol concentrations improve the antioxidant capacity and provide systemic health benefits, including reduced inflammation and enhanced cardiovascular protection.

Furthermore, the rise in the soluble protein content following WD treatment significantly enhanced the functional and nutritional properties of the chickpea flour. As a major protein source in vegetarian and vegan diets [46], chickpeas benefit from an elevated soluble protein fraction, which may also improve their technological characteristics, such as their emulsifying capacity and texture in processed foods. Of particular interest is that this fraction primarily consists of albumins, which are richer in sulfur-containing amino acids than other legume storage proteins [47]. Consequently, their increase under equal-dry-weight conditions suggests meaningful nutritional fortification in chickpeas from WD-exposed plants. This finding aligns with previous studies reporting similar results in chickpeas from plants subjected to foliar WD application (0.2–0.25%, *v*/*v*) [23,24], thereby supporting its role in improving the overall nutritional profile of chickpea seeds.

Despite the increase in the soluble protein content, SDS-PAGE analysis did not reveal substantial alterations in the protein profile of chickpea seeds following WD treatment. This suggests that exposure to WD does not promote an extensive up- or down-regulation of specific proteins, nor a rearrangement of the relative abundance of distinct proteoforms. Instead, the data were reasonably consistent, including when considering the results of soluble protein quantification, with a general increase in the total protein pool, which largely reflected the proteomic composition observed in the seeds from the untreated plants. The only exception to this similarity lay in ten protein spots showing quantitative variation, with MS identification revealing the up-regulation of two legumin isoforms in the seeds from the WD-exposed plants. Conversely, two different vicilin-like protein isoforms and a legumin J-like spot exhibited opposing regulation patterns, suggesting that WD may differentially influence the accumulation of specific globulin proteoforms.

Importantly, the differences observed in the abundance profiles of legumin(-like) and vicilin-like proteins, as well as in the previously discussed flour nutritional properties, cannot be ascribed to environmental and edaphic variability, as all the plants were cultivated simultaneously within the same small, uniformly managed field plot under identical climatic and soil conditions.

Legumin and vicilin are salt-soluble seed storage globulins that are classified based on their sedimentation coefficients, 11S and 7S, respectively [48,49]. Legumin is a hexameric protein rich in sulfur-containing amino acids, while vicilin exists as a trimer stabilized by noncovalent hydrophobic interactions [50]. Despite their overall similar amino acid composition, legumin(-like) proteins contain higher levels of cysteine and methionine residues than vicilin(-like) proteins [47,50,51]. Nonetheless, the observed differences in the relative protein abundance do not suggest a targeted boosting effect of WD on the enrichment of sulfur-rich protein sources, at least within the globulin fraction. It is worth noting, however, that distinct proteoforms are often associated with different functional properties due to variations in their physical and chemical characteristics. In this context, legumin and vicilin have recently garnered attention as effective carriers for the encapsulation and delivery of bioactive compounds, such as folic acid, with promising nutraceutical applications during pregnancy and in the prevention of anemia [52,53]. Moreover, their amino acid composition, particularly the presence of cysteine residues, endows these proteins with the capacity to interact with inorganic minerals via sulfhydryl groups, thereby facilitating mineral stabilization and enhancing their bioavailability [54]. As a result, the observed reduction in Ca, K, P, and Zn may be partially explained by a dilution effect linked to the increased seed biomass, to which both the protein and carbohydrate contents contributed. While the proportional concentration of these minerals decreased, their total amounts may have remained almost unchanged. This hypothesis also opens the discussion to a possible absolute increase in the other tested minerals, whose concentrations remained rather constant. The observed changes in the specific proteoform abundance upon WD treatment may subtly reflect a modulation of the reported functional properties, although further targeted investigations are needed.

Nowadays the incidence of food allergies is rapidly increasing, with approximately 10% of the population affected [55]. Despite chickpea allergens remaining poorly characterized, cross-reactivity has been observed in pea-allergic patients [56,57]. Given that WD treatment induced only minimal alterations in seed proteomic profiles, it is unlikely that any significant impact on the allergic potential of seeds from treated plants would arise, beyond the already-known allergic potential for untreated chickpeas and unless sensitization is linked to an increased concentration of proteins with allergic potential.

## 4. Materials and Methods

### 4.1. Experimental Design

**Figure 5:**
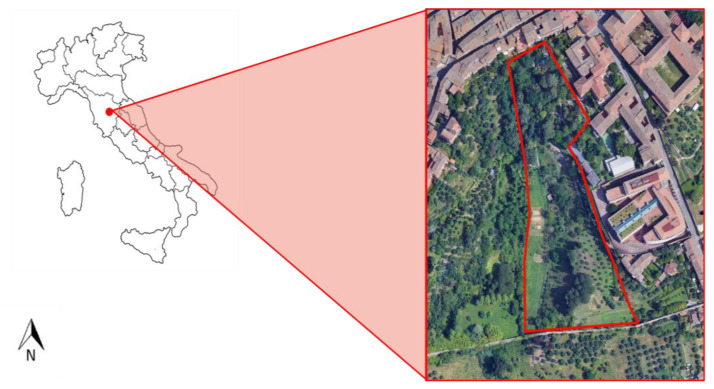
Location of the experiment.

Dried chickpea seeds, supplied by a local farm, were placed in 50 mL tubes and subjected to cold stratification by storing them at 6 °C for 3 days, and then they were sterilized using 3% (*v*/*v*) sodium hypochlorite (NaClO) for 2 min, followed by thorough rinsing with deionized water (dH_2_O). The seeds were then left to germinate in Petri dishes at 15 °C in the dark for one week. The resulting seedlings were transplanted into plastic pots (10 cm × 10 cm × 12 cm) filled with soil and grown for 2 weeks. After this period, eighteen plants were transplanted into the ground in the Botanical Garden of the University of Siena, Italy (43°18′43873″ N, 11°19′48212″ E; Figure 5). Nine of these plants were treated weekly with a soil drenching application of 0.5% (*v*/*v*) of wood distillate, while the remaining nine plants served as the control group and were watered with tap water only. The chosen concentration was based on preliminary tests and supported by the relevant scientific literature. The experiment lasted for 4 months, from February to June 2021. The characteristics of the WD applied in this experiment are reported in Celletti et al. [58] and detailed in Table 4. After the 4-month growth and ripening period, whole plants were harvested and brought to the laboratory for further analysis.

### 4.2. Yield Parameters

Before harvest, the plant height was measured by considering the maximum distance between the ground level and the plant apex. After harvest, the plants were dried at 40 °C for one week, and their biomass was measured. The number of pods and seeds, as well as the seed diameter and weight, were also recorded.

### 4.3. Flour Nutritional Parameters

The collected chickpea seeds were ground using a professional laboratory mixer (IKA A10) to obtain uniform flours for nutrient analyses.

#### 4.3.1. Mineral Elements

The mineral content was determined using the method described by Fedeli et al. [59], using a portable X-ray fluorescence (XRF) device. Approximately 1 g of chickpea flour was placed in a plastic cup, which was then inserted into the designated compartment of the instrument. The content of Al, Ca, Cu, Fe, K, Mn, P, S, and Zn was measured in Geochem mode, with an acquisition time of 20 s per beam, for a total of three beams per analysis. The accuracy of the analysis was validated using 14 certified plant matrices, as reported by Fedeli et al. [60]. The results are expressed as mg of element per kg of dry weight.

#### 4.3.2. Soluble Sugars

The content of soluble sugars (i.e., glucose, sucrose, and fructose) was determined following the procedure described by Fedeli et al. [23]. Approximately 100 mg of chickpea flour was homogenized in 2 mL of dH_2_O and centrifuged at 3000× *g* for 5 min. The supernatant was filtered through a 0.45 μm syringe filter and directly analyzed by HPLC (Waters 600 system, Waters Corporation, Milford, MA, USA) using a Waters 2410 refractive index detector. Sugar separation was achieved using deionized water as the mobile phase, eluted at 0.5 mL/min, and a Waters Sugar-Pak I ion-exchange column (6.5 mm × 300 mm) maintained at 90 °C using an external temperature controller (Waters Column Heater Module). Quantification was performed using calibration curves prepared by dissolving analytical sugars (Sigma-Aldrich, St. Louis, MO, USA) in dH_2_O at concentrations of 0.1–20 mg/mL.

#### 4.3.3. Starch

The content of starch was determined following the procedure described by Lamaro et al. [61]. Approximately 50 mg of chickpea flour was homogenized in 2 mL of pure dimethyl sulfoxide (DMSO). Then, 0.5 mL of 8 M HCl was added, and the samples were placed in a ventilated oven for 30 min at 60 °C. After cooling, 0.5 mL of 8 M NaOH and 7 mL of dH_2_O were added. The samples were then centrifuged at 2100× *g* for 5 min, and 0.5 mL of the supernatant was mixed with 2.5 mL of Lugol’s solution. After 15 min, the samples were measured at 605 nm using a UV-VIS spectrophotometer (Agilent 8453, Agilent Technologies, Santa Clara, CA, USA). Quantification was carried out using a calibration curve (10–400 μg/mL) prepared with pure starch (Sigma-Aldrich, St. Louis, MO, USA).

#### 4.3.4. Total Antioxidant Power

The total antioxidant power was determined following the procedure described by Fedeli et al. [62]. Approximately 100 mg of chickpea flour was homogenized in 2 mL of 80% (*v/v*) ethanol and centrifuged at 3000× *g* for 5 min. An aliquot of the supernatant (200 μL) was added to 1 mL of 2,2-diphenyl-1-picrylhydrazyl (DPPH) solution, and the samples were kept in the dark for 1 h. A blank (200 μL of 80% (*v/v*) ethanol in 1 mL of 80% (*v/v*) methanol) and a control (200 μL of 80% (*v/v*) ethanol in 1 mL of the DPPH solution) were also prepared for comparison. Finally, the absorbance was measured at 517 nm using a UV-Vis spectrophotometer (Agilent 8453). The results were expressed as a percentage of the antiradical activity (ARA%), calculated using the following formula:

ARA%=100×[1−(sample absorbance/control absorbance)]

#### 4.3.5. Total Polyphenols

The content of the total polyphenols (TPC) was determined following the procedure described by Fedeli et al. [63]. Approximately 100 mg of the chickpea flour was homogenized in 4 mL of 70% (*v/v*) acetone and centrifuged at 2100× *g* for 5 min. The supernatant (0.5 mL) was mixed with 3 mL of dH_2_O, 0.125 mL of Folin–Denis’s reagent (Sigma-Aldrich, St. Louis, MO, USA), 0.750 mL of saturated Na_2_CO_3_, and 0.950 mL of deionized water. The samples were placed in an oven at 37 °C for 30 min, then centrifuged at 2100× *g* for 5 min, and the absorbance was measured at 765 nm using a UV-Vis spectrophotometer (Agilent 8453). Quantification was performed using a calibration curve (5–20 μg/mL) with gallic acid (Sigma-Aldrich) used as the standard. The results were expressed as mg of gallic acid equivalent per gram of dry weight (mg GAE/g dw).

#### 4.3.6. Total Soluble Proteins

The content of the total soluble proteins was determined following the procedure described by Bradford [64]. Approximately 50 mg of chickpea flour was homogenized in 5 mL of dH_2_O and centrifuged at 2100× *g* for 5 min. Then, 0.4 mL of the supernatant was added to 1.6 mL of Bradford reagent (Sigma-Aldrich). The soluble protein content was determined using a UV-Vis spectrophotometer (Agilent 8453) by measuring the absorbance at 595 nm. Quantification was performed using a calibration curve, with bovine serum albumin (BSA) (Sigma-Aldrich) used as the standard, at concentrations of 20–80 μg/mL. The results were expressed as mg of BSA equivalent per gram of dry weight (mg BSA eq/g dw).

#### 4.3.7. Statistical Analysis for Yield, Biochemical, Micro-, and Macronutrient Parameters

Since the data approached a normal distribution (Shapiro–Wilk test, *p* > 0.05), the results are reported as the mean ± the standard error, and statistical comparisons between the control and WD-treated samples were run using Student’s *t*-test with a significance level of *p* < 0.05. Statistical analysis was carried out using R software 4.4.1 [65].

### 4.4. Protein Sample Extraction for Proteomics Analyses

Six dehydrated chickpeas from WD-treated plants and six from untreated ones, each sourced from a different plant, were individually processed through mechanical grinding to obtain separate coarse flours. Two hundred µg from each flour sample was individually rehydrated, at 4 °C and for 6 h, in a protein extraction buffer containing 9 M urea, 4% (*w*/*v*) 3-[(3-cholamidopropyl) dimethylammonia]-1-propanesulfonate hydrate (CHAPS), 40 mM Tris, and 65 mM dithioerythritol (DTE). After adding a single pre-chilled stainless-steel bead (5 mm diameter) (Qiagen, Hilden, Germany) to each sample, the tubes were then placed in a pre-chilled cold TissueLyser Reaction-Tube Holder (Qiagen) and secured on the Tissue Lyser II platform (Qiagen) for homogenization. This was achieved by performing 6 × 1 min cycles at 9.3 kHz (1200 oscillations per minute) and resting the samples on ice in between. The extracts were then cleared by centrifugation at 21,000× *g* for 10 min at 4 °C, and the recovered supernatants were precipitated overnight in 1:4 cold acetone at −20 °C. After sample/acetone centrifugation at 15,000× *g* for 15 min at 4 °C, the pellets were re-suspended in the same buffer used for sample homogenization, and the insolubilized material was discarded, as pellets, by performing a 21,000× *g* centrifugation step for 15 min at 4 °C. Sample concentration was estimated using the Bradford assay [64] and the aliquots stored at −80 °C until use.

### 4.5. Proteomic Analyses

The 12 flour protein samples were separated by 2DE, according to the procedure described by Bianchi et al. [66] (2022). For every analytical 2DE run, 60 μg of protein sample, along with 0.2% (*v*/*v*) of pH 3–10 carrier ampholytes, was loaded via cathodic cup-loading onto isoelectrofocusing (IEF) strips, which had a pH 3–10 non-linear gradient and were 18 cm in length (Cytiva, formerly GE Healthcare, Marlborough, MA, USA), using an Ettan IPGphor system (Cytiva). For each MS-preparative 2DE run, 600 µg was loaded, along with 2% (*v*/*v*) pH 3–10 carrier ampholytes, onto the same strip type. IEF was carried out on an Ettan IPGphor Manifold (Cytiva) by applying the following voltage settings at 16 °C: 200 V for 8 h, a transition from 200 V to 3500 V over 2 h, 3500 V for 2 h, a transition from 3500 V to 5000 V over 2 h, 5000 V for 3 h, a gradient to 8000 V over 1 h, and maintenance at 8000 V up to a total of 95,000 V h. Then, the strips were equilibrated for 12 min in 6 M urea, 30% (*v*/*v*) glycerol, 2% (*w*/*v*) sodium dodecyl sulfate (SDS), 0.05 M Tris–HCl at pH 6.8, and 2% (*w*/*v*) DTE; and for a further 5 min in 6 M urea, 30% (*v*/*v*) glycerol, 2% (*w*/*v*) SDS, 0.05 M Tris–HCl at pH 6.8, 2.5% (*w*/*v*) iodoacetamide, and a trace amount of bromophenol blue. The second dimension was carried out at 9 °C on 9–16% polyacrylamide linear-gradient gels (18 cm × 20 cm × 1.5 mm) at 40 mA/gel. The analytical gels were stained using ammoniacal silver nitrate staining [67], while the MS-preparative gels were stained according to the procedure described by Gharadaghi et al. [68]. The gel images were digitalized using the Image Scanner III and LabScan 6.0 software (GE Healthcare, Chicago, IL, USA) and then analyzed using Melanie™ Classic 9 (SIB Swiss Institute of Bioinformatics, Geneva, Switzerland). The relative volume percentage values (%Vol), corresponding to the ratio between the optical density of a single spot (volume) and the total volume of spots present in the same gel and expressed as a percentage, were exported for statistical analysis.

A statistical comparison between the flours of chickpeas from WD-treated and not-treated plants was performed by using a linear model for the assessment of differential abundances (limma R package v.4.4) [68]. Protein differences presenting a *p* ≤ 0.05 and an FC ≥ 2 were considered relevant and represented in a heatmap plot, where their clustering was obtained using Ward’s method involving Euclidean distances. A variance–covariance analysis was performed using principal component analysis (PCA) on the %Vol values of significantly differing proteins. The statistical analyses and corresponding figures were obtained by using R software 4.4.1 [65].

### 4.6. Mass Spectrometry

Statistically significant differential spots were manually cut out from the MS-preparative gels, destained [69], partially dehydrated in 5 mM ammonium bicarbonate and a 50% (*v*/*v*) acetonitrile solution, and then completely dehydrated in 100% acetonitrile (ACN). Tryptic peptides, obtained through overnight trypsin digestion at 37 °C, were placed on a MALDI target, air-dried, and covered with a matrix solution of 5 mg/mL α-cyano-4-hydroxycinnamic acid (CHCA) in 50% (*v*/*v*) ACN and 0.5% (*v*/*v*) trifluoroacetic acid (TFA). The mass spectrometry spectra were acquired using an UltrafleXtreme™ MALDI-TOF/TOF mass spectrometer (Bruker Daltonics, Billerica, MA, USA), equipped with a 200 Hz smartbeam™ I laser (Bruker) in the positive reflector mode, and according to the following parameters: 80 ns of delay; ion source 1: 25 kV; ion source 2: 21.75 kV; lens voltage: 9.50 kV; reflector 1 voltage: 26.30 kV; and reflector 2 voltage: 14.00 kV. The applied laser wavelength and frequency were 353 nm and 100 Hz, respectively, and the percentage was set to 50%. The MS spectra were processed using FlexAnalysis 3.0 software (Bruker Daltonics), using peptides produced through trypsin autoproteolysis as internal standards for calibration. Common contaminants, such as matrix-related ions, trypsin autolysis, and keratin peaks, were removed from the resulting mass lists, and protein identification was carried out via a Peptide Mass Fingerprinting (PMF) search using MASCOT (Matrix Science Ltd., London, UK). The PMF search was performed by setting the following parameters: *Cicer arietinum* as the taxonomy, with its proteome -UP000087171 - uploaded onto an in-house Mascot server and used as the searching database, 50 ppm as the mass tolerance, one admissible missed cleavage site, the carbamidomethylation of cysteine as fixed modification, and the oxidation of methionine as variable modification. Mass spectrometry analyses were performed using the Molsys Technology Platform (http://molsys.dbcf.unisi.it; accessed on 24 February 2025). Only protein identifications with a *p*-value ≤ 0.03 (referred to as “expected”), a minimum of 4 matched peptides, and a minimum Mascot score of 60 were considered.

## 5. Conclusions

The present work set out to explore whether soil drenching with WD could enhance the chickpea yield and seed nutritional quality.

The treatment significantly improved the productivity by enhancing the total plant biomass, mostly due to reproductive growth rather than vegetative biomass, as indicated by the minimal change in plant height, along with the number of pods and seeds, and the seed size. Higher levels of soluble proteins and polyphenols and a greater antioxidant capacity were observed. Also starch, sucrose, and fructose concentrations increased, while glucose levels declined, potentially contributing to a lower GI. Although their proportional content decreased, Ca, K, P, and Zn remained within acceptable ranges. Proteomic analysis revealed only minor changes restricted to the globulin fraction, indicating that the overall chickpea protein profile and nutritional safety were preserved. In conclusion, soil drenching with WD is a promising and sustainable biostimulant for use in chickpea cultivation.

## Figures and Tables

**Table 1:** Yield parameters (mean ± standard error) of chickpea plants (*n* = 9) treated (WD) or not treated (CTRL) with 0.5% (*v/v*) wood distillate. * = statistically significant (*p* < 0.05) difference.

Yield Parameters	CTRL	WD
Plant height (cm)	62 ± 13	75 ± 4
Plant biomass (g) *	25 ± 8	61 ± 8
Number of pods *	31 ± 5	77 ± 18
Number of seeds *	21 ± 4	52 ± 12
Seed diameter (mm) *	6.75 ± 0.15	7.14 ± 0.08
Seed weight (mg) *	0.28 ± 0.02	0.35 ± 0.02

**Table 2:** Content of mineral elements (mean ± standard error) of chickpea plants (*n* = 9) treated (WD) or not treated (CTRL) with 0.5% (*v/v*) wood distillate. * = statistically significant (*p* < 0.05) difference.

Mineral Element (mg kg^−1^)	CTRL	WD
Al	340 ± 35	284 ± 40
Ca *	416 ± 32	287 ± 7
Cu	5.2 ± 0.4	4.8 ± 0.4
Fe	24 ± 1	22 ± 1
K *	5966 ± 98	5268 ± 127
Mn	18 ± 1	16 ± 1
P *	2707 ± 40	2568 ± 30
S	977 ± 25	938 ± 43
Zn *	29 ± 1	25 ± 1

**Table 3:** MS-identified protein spots differing significantly among control and WD-treated plant seeds.

Spot n. ^a^	UniprotKB ^b^	UniProtKB ID ^c^	Statistics ^d^			Mascot Search ^e^				
			*p*-Value	Ctrl	WD	pI-MW	S	C	MP	E
1	Vicilin-like	A0A1S2XQ88_CICAR	3.93 × 10^−2^	222.11 ± 70.13	63.10 ± 34.32	6.10-51,227	91	18	7/13	2.7 × 10^−5^
2	Legumin	LEG_CICAR	3.93 × 10^−2^	188.24 ± 36.03	317.08 ± 50.85	6.20-56,672	60	9	5/11	0.031
3	Legumin	LEG_CICAR	1.55 × 10^−2^	104.76 ± 21.03	234.54 ± 41.52	6.20-56,672	62	9	4/5	0.017
4	Vicilin-like	A0A1S2XQ88_CICAR	3.93 × 10^−2^	450.50 ± 79.44	257.03 ± 51.13	6.10-51,227	70	12	5/8	0.003
7	Legumin J-like	A0A1S2XVG1_CICAR	1.55 × 10^−2^	937.95 ± 208.57	300.54 ± 111.15	5.50-60,847	82	20	8/27	0.0002

^a^ Spot numbers correspond to those used in Figure 2 to indicate protein spot differences; ^b^ UniProtKB-recommended protein name; ^c^ UniProtKB Identifier; ^d^ statistics reports: statistical significance of differential abundance between Ctrl and WD groups, with corresponding spot abundance (%Vol) means and standard deviation; ^e^ Mascot search results: theoretical isoelectric point (pI) and molecular weight (MW - Da), identification score (S), coverage (C), number of experimentally detected peptides, out of the total number of detected peptides, matching the theoretical sequence, (MP), and expected (E)-value (E). Protein differences were considered significant with FC ≥ 2 and *p* ≤ 0.05.

**Table 4:** Main physical and chemical characteristics of the wood distillate used in this study.

Parameter	Value	Measurement Method
TOC (% DW)	58.03	CHNS elemental analysis
TN (% DW)	1.06	CHNS elemental analysis
H (% DW)	7.27	CHNS elemental analysis
S (% DW)	0.07	CHNS elemental analysis
pH	4	UNI EN ISO 10523:2012
Density (g mL^−1^)	1.05	
Flash point (°C)	>60	ASTM D6450-16a
Total organic compounds (g L^−1^)	33.8	
Acidity (mg L^−1^)	1289	APAT CNR IRSA 2010 B Man 29 2003
Organic acids (mg L^−1^)	32.3	
Acetic acid (mg L^−1^)	21.5	
Polyphenols (g L^−1^)	24.5	
Phenols (g L^−1^)	3	
PCBs (mg L^−1^)	<0.2	CNR IRSA 24b Q 64 Vol 3 1988
Hydrocarbons, C < 12 (mg L^−1^)	<0.1	EPA 5021A 2014 + EPA 8015D 2003
Hydrocarbons, C10–C40 (mg L^−1^)	<0.1	UNI EN ISO 9377-2:2002
**16 US-EPA PAHs (mg L^−1^)**		EPA 3550C 2007 + EPA 8310 1986
Acenaphthene	<0.05	
Acenaphthylene	<0.05	
Anthracene	<0.05	
Benzo[a]anthracene	<0.05	
Benzo[a]pyrene	<0.05	
Benzo[b]fluoranthene	<0.05	
Benzo[g,h,i]perylene	<0.05	
Benzo[k]fluoranthene	<0.05	
Chrysene	<0.05	
Dibenz[a,h]anthracene	<0.05	
Fluoranthene	<0.05	
Fluorene	<0.05	
Indeno[1,2,3-cd]pyrene	<0.05	
Naphthalene	<0.05	
Phenanthrene	<0.05	
Pyrene	<0.05	
**Macronutrients (mg L^−1^)**		Alkaline melting + ICP-MS analysis
Ca	325.50	
K	23.49	
Mg	6.79	
P	7.28	
**Micronutrients (mg L^−1^)**		Alkaline melting + ICP-MS analysis
Cu	0.18	
Fe	21.16	
Mn	0.58	
Mo	0.0007	
Zn	3.22	
**Other nutrients**		Alkaline melting + ICP-MS analysis
Al	1.96	
Ba	0.06	
Cr	0.03	
Na	103.59	

**TOC**: total organic carbon. **TN**: total nitrogen. **PCBs**: polychlorinated biphenyls. **16 US-EPA PAHs**: list of 16 priority polycyclic aromatic hydrocarbons as classified by United States Environmental Protection Agency. **Al**: aluminum; **Ba**: barium; **C**: carbon; **Ca**: calcium; **Cr**: chromium; **Cu**: copper; **Fe**: iron; **K**: potassium; **Mg**: magnesium; **Mn**: manganese; **Mo**: molybdenum; **N**: nitrogen; **Na**: sodium; **Zn**: zinc.

## Data Availability

The authors declare that they have no known competing financial interests or personal relationships that could have appeared to influence the work reported in this paper.

## Supplementary Materials

The following supporting information can be downloaded at https://www.mdpi.com/article/10.3390/plants14132046/s1. Figure S1: Digital images of six control chickpea gels (A–F) and of six gels from chickpeas harvested from WD soil drenched plants (G–L), used for differential analysis. Each gel corresponds to the protein pattern of a single seed, and all analyzed seeds were collected from different pods and plants.

## References

[1] Jukanti A.K., Gaur P.M., Gowda C.L.L., Chibbar R.N. (2012). Nutritional quality and health benefits of chickpea (*Cicer arietinum* L.): A review. Br. J. Nutr..

[2] Floret C., Monnet A.F., Micard V., Walrand S., Michon C. (2023). Replacement of animal proteins in food: How to take advantage of nutritional and gelling properties of alternative protein sources. Crit. Rev. Food Sci. Nutr..

[3] Quintieri L., Nitride C., De Angelis E., Lamonaca A., Pilolli R., Russo F., Monaci L. (2023). Alternative protein sources and novel foods: Benefits, food applications and safety issues. Nutrients.

[4] Koneswaran G., Nierenberg D. (2008). Global farm animal production and global warming: Impacting and mitigating climate change. Environ. Health Perspect..

[5] McMichael A.J., Powles J.W., Butler C.D., Uauy R. (2007). Food, livestock production, energy, climate change, and health. Lancet.

[6] Lal R. (2017). Improving soil health and human protein nutrition by pulses-based cropping systems. Adv. Agron..

[7] Mrabet R. (2023). Sustainable agriculture for food and nutritional security. Sustainable Agriculture and the Environment.

[8] Nadathur S., Wanasundara J.P., Scanlin L. (2024). Feeding the globe nutritious food in 2050: Obligations and ethical choices. Sustainable Protein Sources.

[9] Hossain M.E., Shahrukh S., Hossain S.A. (2022). Chemical fertilizers and pesticides: Impacts on soil degradation, groundwater, and human health in Bangladesh. Environmental Degradation: Challenges and Strategies for Mitigation.

[10] Tripathi S., Srivastava P., Devi R.S., Bhadouria R. (2020). Influence of synthetic fertilizers and pesticides on soil health and soil microbiology. Agrochemicals Detection, Treatment and Remediation.

[11] Prashar P., Shah S. (2016). Impact of fertilizers and pesticides on soil microflora in agriculture. Sustainable Agriculture Reviews.

[12] Grewal A., Abbey L., Gunupuru L.R. (2018). Production, prospects and potential application of pyroligneous acid in agriculture. J. Anal. Appl. Pyrolysis.

[13] Lu S.N., Zhou L., Wang L.J., Liu L., Zhang H.M. (2023). Research progress on preparation of wood vinegar and its application in agriculture. J. Shanxi Agric. Sci..

[14] Wei Q., Ma X., Zhao Z., Zhang S., Liu S. (2010). Antioxidant activities and chemical profiles of pyroligneous acids from walnut shell. J. Anal. Appl. Pyrolysis.

[15] Gomez J.P., Velez J.P.A., Pinzon M.A., Arango J.A.M., Muriel A.P. (2021). Chemical characterization and antiradical properties of pyroligneous acid from a preserved bamboo, *Guadua angustifolia* Kunth. Braz. Arch. Biol. Technol..

[16] Becagli M., Arduini I., Cantini V., Cardelli R. (2022). Soil and foliar applications of wood distillate differently affect soil properties and field bean traits in preliminary field tests. Plants.

[17] Bianchi G., Fedeli R., Mariotti L., Pisuttu C., Nali C., Pellegrini E., Loppi S. (2024). Foliar application of wood distillate protects basil plants against ozone damage by preserving membrane integrity and triggering antioxidant mechanisms. Agronomy.

[18] Mungkunkamchao T., Kesmala T., Pimratch S., Toomsan B., Jothityangkoon D. (2013). Wood vinegar and fermented bioextracts: Natural products to enhance growth and yield of tomato (*Solanum lycopersicum* L.). Sci. Hortic..

[19] Akley E.K., Ampim P.A., Obeng E., Sanyare S., Yevu M., Owusu Danquah E., Seidu A.F. (2023). Wood vinegar promotes soil health and the productivity of cowpea. Agronomy.

[20] Lau S.E., Lim L.W.T., Hamdan M.F., Chan C., Saidi N.B., Ong-Abdullah J., Tan B.C. (2025). Enhancing plant resilience to abiotic stress: The power of biostimulants. Phyton.

[21] Zhu K., Gu S., Liu J., Luo T., Khan Z., Zhang K., Hu L. (2021). Wood vinegar as a complex growth regulator promotes the growth, yield, and quality of rapeseed. Agronomy.

[22] Ahadiyat Y.R., Hadi S.N., Herliana O. (2018). Application of wood vinegar coconut shell and NPK fertilizer to maintain sustainable agriculture of upland rice production. J. Degrad. Min. Lands Manag..

[23] Fedeli R., Marotta L., Frattaruolo L., Panti A., Carullo G., Fusi F., Loppi S. (2023). Nutritionally enriched tomatoes (*Solanum lycopersicum* L.) grown with wood distillate: Chemical and biological characterization for quality assessment. J. Food Sci..

[24] Carril P., Bianchi E., Cicchi C., Coppi A., Dainelli M., Gonnelli C., Colzi I. (2023). Effects of wood distillate (pyroligneous acid) on the yield parameters and mineral composition of three leguminous crops. Environments.

[25] Noel R., Schueller M.J., Ferrieri R.A. (2024). Radiocarbon flux measurements provide insight into why a pyroligneous acid product stimulates plant growth. Int. J. Mol. Sci..

[26] Simkin A.J., Kapoor L., Doss C.G.P., Hofmann T.A., Lawson T., Ramamoorthy S. (2022). The role of photosynthesis related pigments in light harvesting, photoprotection and enhancement of photosynthetic yield in planta. Photosynth. Res..

[27] Melis A. (2009). Solar energy conversion efficiencies in photosynthesis: Minimizing the chlorophyll antennae to maximize efficiency. Plant Sci..

[28] Shah L., Yahya M., Shah S.M.A., Nadeem M., Ali A., Ali A., Ma C. (2019). Improving lodging resistance: Using wheat and rice as classical examples. Int. J. Mol. Sci..

[29] Nandal V., Solanki M. (2021). The Zn as a vital micronutrient in plants. J. Microbiol. Biotechnol. Food Sci..

[30] Schachtman D.P., Reid R.J., Ayling S.M. (1998). Phosphorus uptake by plants: From soil to cell. Plant Physiol..

[31] Vandemark G.J., Grusak M.A., McGee R.J. (2018). Mineral concentrations of chickpea and lentil cultivars and breeding lines grown in the US Pacific Northwest. Crop J..

[32] Iqbal A., Ateeq N., Khalil I.A., Perveen S., Saleemullah S. (2006). Physicochemical characteristics and amino acid profile of chickpea cultivars grown in Pakistan. J. Foodserv..

[33] Zia-Ul-Haq M., Iqbal S., Ahmad S., Imran M., Niaz A., Bhanger M.I. (2007). Nutritional and compositional study of desi chickpea (*Cicer arietinum* L.) cultivars grown in Punjab, Pakistan. Food Chem..

[34] Englyst K.N., Vinoy S., Englyst H.N., Lang V. (2003). Glycaemic index of cereal products explained by their content of rapidly and slowly available glucose. Br. J. Nutr..

[35] Foster-Powell K., Miller J.B. (1995). International tables of glycemic index. Am. J. Clin. Nutr..

[36] Singh M., Manickavasagan A., Shobana S., Mohan V. (2021). Glycemic index of pulses and pulse-based products: A review. Crit. Rev. Food Sci. Nutr..

[37] Sun L., Miao M. (2020). Dietary polyphenols modulate starch digestion and glycaemic level: A review. Crit. Rev. Food Sci. Nutr..

[38] Dhital S., Warren F.J., Butterworth P.J., Ellis P.R., Gidley M.J. (2017). Mechanisms of starch digestion by α-amylase—Structural basis for kinetic properties. Crit. Rev. Food Sci. Nutr..

[39] Fedeli R., Vannini A., Guarnieri M., Monaci F., Loppi S. (2022). Bio-based solutions for agriculture: Foliar application of wood distillate alone and in combination with other plant-derived corroborants results in different effects on lettuce (*Lactuca sativa* L.). Biology.

[40] Lu X., Jiang J., He J., Sun K., Sun Y. (2019). Pyrolysis of *Cunninghamia lanceolata* waste to produce wood vinegar and its effect on the seeds germination and root growth of wheat. BioResources.

[41] Rana A., Samtiya M., Dhewa T., Mishra V., Aluko R.E. (2022). Health benefits of polyphenols: A concise review. J. Food Biochem..

[42] Scarpa E.S., Antonelli A., Balercia G., Sabatelli S., Maggi F., Caprioli G., Micucci M. (2024). Antioxidant, anti-inflammatory, anti-diabetic, and pro-osteogenic activities of polyphenols for the treatment of two different chronic diseases: Type 2 diabetes mellitus and osteoporosis. Biomolecules.

[43] Trinidad T.P., Mallillin A.C., Loyola A.S., Sagum R.S., Encabo R.R. (2010). The potential health benefits of legumes as a good source of dietary fibre. Br. J. Nutr..

[44] Maphosa Y., Jideani V.A. (2017). The role of legumes in human nutrition. Functional Food—Improve Health Through Adequate Food.

[45] Martín-Cabrejas M.Á. (2019). Legumes: Nutritional Quality, Processing and Potential Health Benefits.

[46] Boukid F. (2021). Chickpea (*Cicer arietinum* L.) protein as a prospective plant-based ingredient: A review. Int. J. Food Sci. Technol..

[47] Di Francesco A., De Santis M.A., Lanzoni A., Pittalà M.G.G., Saletti R., Flagella Z., Cunsolo V. (2024). Mass spectrometry characterization of the SDS-PAGE protein profile of legumins and vicilins from chickpea seed. Foods.

[48] Boye J.I., Zare F., Pletch A. (2010). Pulse proteins: Processing, characterization, functional properties and applications in food and feed. Food Res. Int..

[49] Day L. (2013). Proteins from land plants—Potential resources for human nutrition and food security. Trends Food Sci. Technol..

[50] Grasso N., Lynch N.L., Arendt E.K., O’Mahony J.A. (2021). Chickpea protein ingredients: A review of composition, functionality, and applications. Compr. Rev. Food Sci. Food Saf..

[51] Shevkani K., Singh N., Chen Y., Kaur A., Yu L. (2019). Pulse proteins: Secondary structure, functionality and applications. J. Food Sci. Technol..

[52] Fang C., Kanemaru K., Carvalho W.S.P., Fruehauf K.R., Zhang S., Das P.P., Serpe M.J. (2024). Self-assembled poloxamer-legumin/vicilin nanoparticles for the nanoencapsulation and controlled release of folic acid. Int. J. Biol. Macromol..

[53] Clare G., Simões P., Costa B.F., Durães L. (2024). Dual-function starch aerogels: Nutraceutical carriers for iron and folic acid delivery. J. Drug Deliv. Sci. Technol..

[54] Brokesh A.M., Gaharwar A.K. (2020). Inorganic biomaterials for regenerative medicine. ACS Appl. Mater. Interfaces.

[55] Sicherer S.H., Sampson H.A. (2018). Food allergy: A review and update on epidemiology, pathogenesis, diagnosis, prevention, and management. J. Allergy Clin. Immunol..

[56] Abu Risha M., Rick E.M., Plum M., Jappe U. (2024). Legume allergens pea, chickpea, lentil, lupine and beyond. Curr. Allergy Asthma Rep..

[57] Wangorsch A., Kulkarni A., Jamin A., Spiric J., Bräcker J., Brockmeyer J., Scheurer S. (2020). Identification and characterization of IgE-reactive proteins and a new allergen (Cic a 1.01) from chickpea (*Cicer arietinum*). Mol. Nutr. Food Res..

[58] Celletti S., Fedeli R., Ghorbani M., Aseka J.M., Loppi S. (2023). Exploring sustainable alternatives: Wood distillate alleviates the impact of bioplastic in basil plants. Sci. Total Environ..

[59] Fedeli R., Di Lella L.A., Loppi S. (2024). Suitability of XRF for routine analysis of multi-elemental composition: A multi-standard verification. Methods Protoc..

[60] Fedeli R., Zhatkanbayeva Z., Loppi S. (2025). Wood Distillate as a Solution for Growing Crops Under Water Deficiency. Crops.

[61] Lamaro G.P., Tsehaye Y., Girma A., Vannini A., Fedeli R., Loppi S. (2023). Evaluation of yield and nutraceutical traits of orange-fleshed sweet potato storage roots in two agro-climatic zones of northern Ethiopia. Plants.

[62] Fedeli R., Cruz C., Loppi S., Munzi S. (2024). Hormetic effect of wood distillate on hydroponically grown lettuce. Plants.

[63] Fedeli R., Vannini A., Djatouf N., Celletti S., Loppi S. (2024). Can lettuce plants grow in saline soils supplemented with biochar?. Heliyon.

[64] Bradford M.M. (1976). A rapid and sensitive method for the quantitation of microgram quantities of protein utilizing the principle of protein-dye binding. Anal. Biochem..

[65] R Core Team (2024). R: A Language and Environment for Statistical Computing.

[66] Bianchi L., Casini S., Vantaggiato L., Di Noi A., Carleo A., Shaba E., Caliani I. (2022). A novel ex vivo approach based on proteomics and biomarkers to evaluate the effects of chrysene, MEHP, and PBDE-47 on loggerhead sea turtles (*Caretta caretta*). Int. J. Environ. Res. Public Health.

[67] Oakley B.R., Kirsch D.R., Morris N.R. (1980). A simplified ultrasensitive silver stain for detecting proteins in polyacrylamide gels. Anal. Biochem..

[68] Ritchie M.E., Phipson B., Wu D., Hu Y., Law C.W., Shi W., Smyth G.K. (2015). limma powers differential expression analyses for RNA-sequencing and microarray studies. Nucleic Acids Res..

[69] Gharahdaghi F., Weinberg C.R., Meagher D.A., Imai B.S., Mische S.M. (1999). Mass spectrometric identification of proteins from silver-stained polyacrylamide gel: A method for the removal of silver ions to enhance sensitivity. Electrophoresis.

