# Supplementary material for: Soil Drenching with Wood Distillate Modifies the Nutritional Properties of Chickpea (Cicer arietinum L.) Seeds by Increasing the Protein Content and Inducing Targeted Changes in the Proteomic Profile
Source: Plants (Basel). 2025 Jul 3;14(13):2046. doi: 10.3390/plants14132046 (PMC12252005; doi:10.3390/plants14132046)
Supplement: Supplementary file 1 [file plants-14-02046-s001.zip › plants-3715986-supplementary.pdf]

Spot 1 → Vicilin like

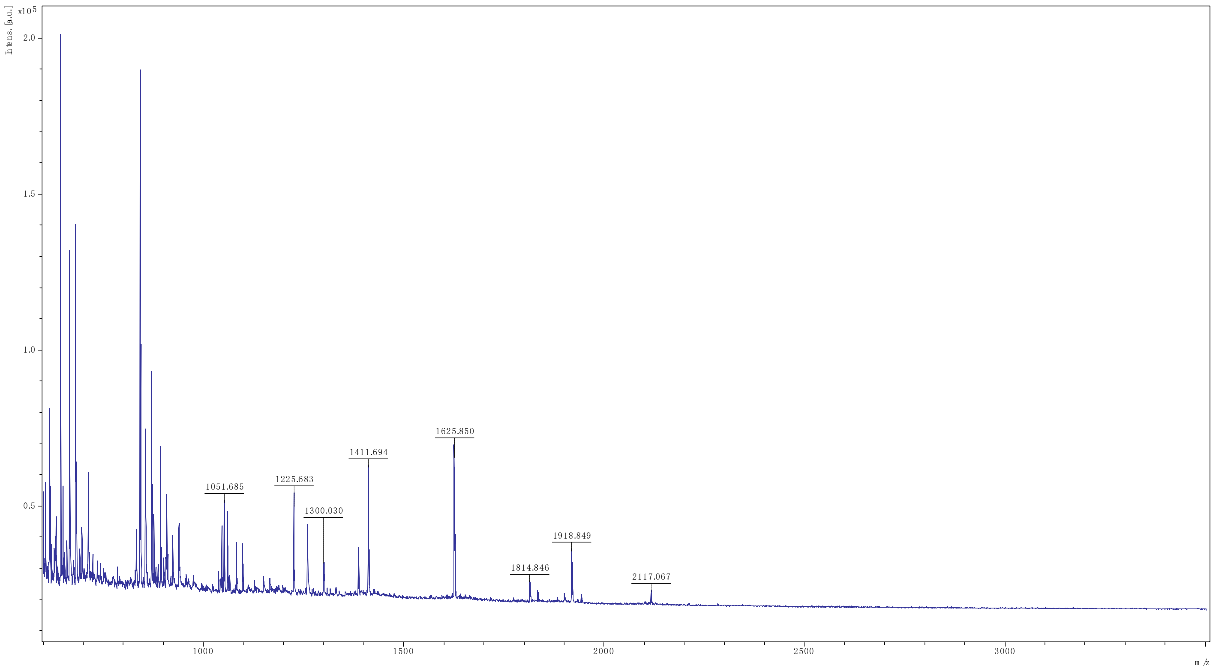

m/z

1045.564

1051.685

1081.996

1097.963

1225.683

1259.705

1300.030

1386.708

1411.694

1625.850

1814.846

1918.849

2117.067

Spot 2 → Legumin

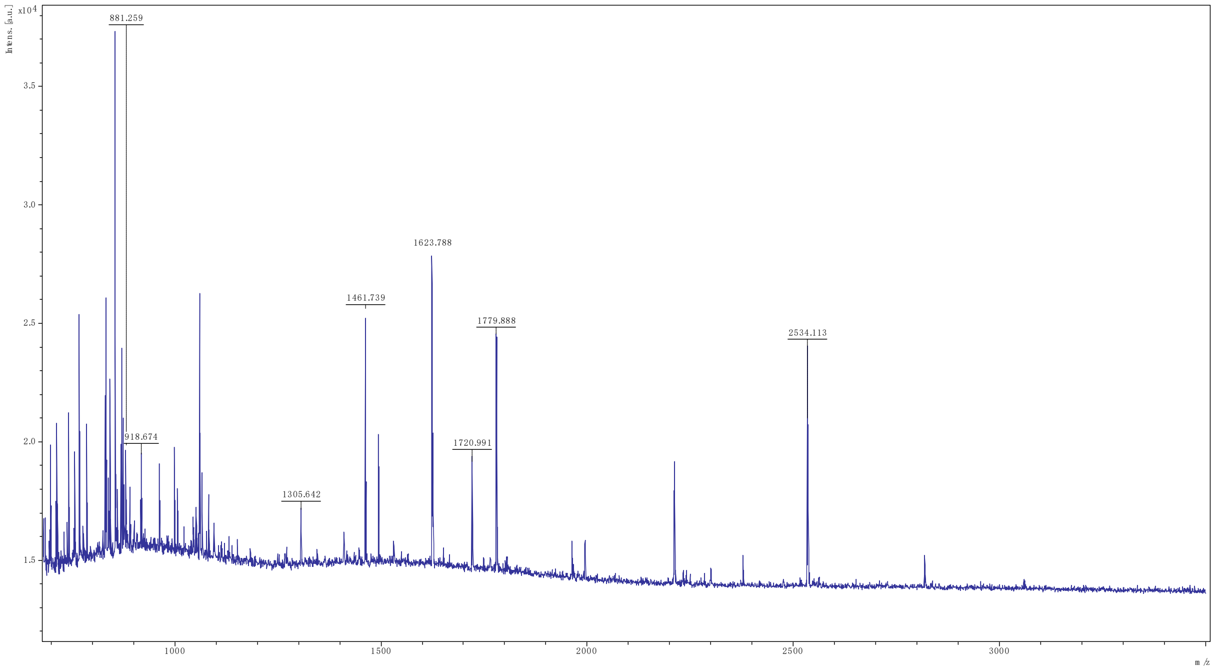

m/z

881.259

918.674

962.698

999.556

1006.726

1305.642

1461.739

1493.829

1623.788

1720.991

1779.888

2534.113

Spot 3 → Legumin

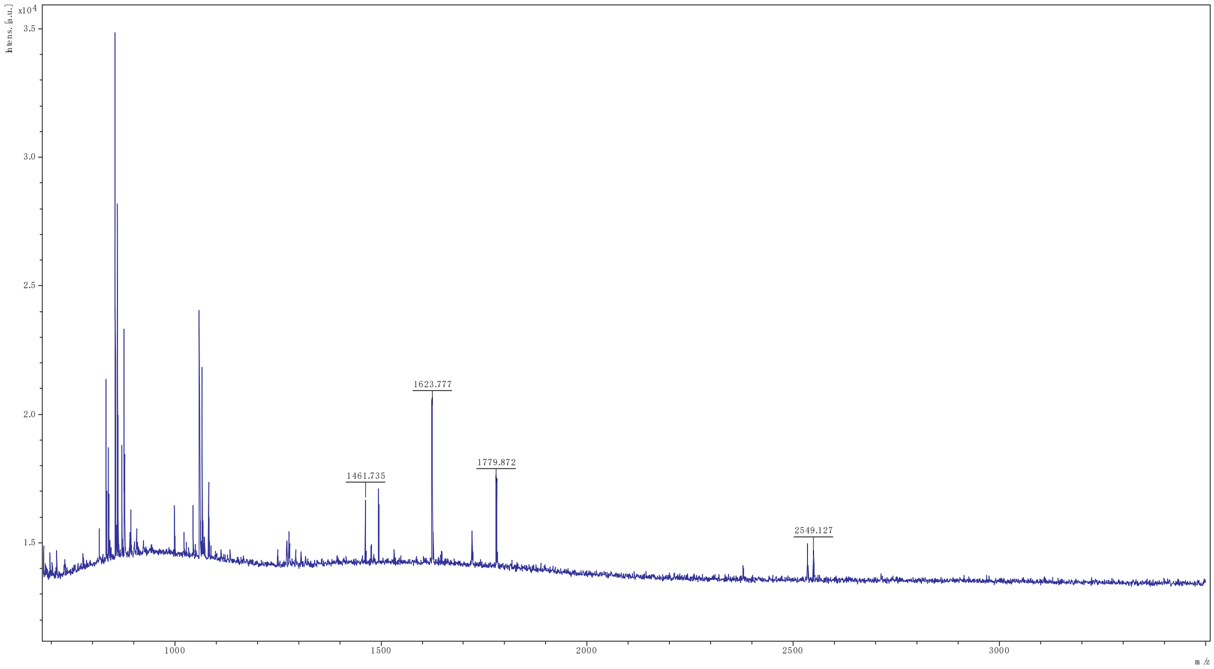

m/z

1461.735

1623.777

1779.872

2534.100

2549.127

Spot 4 → Vicilin like

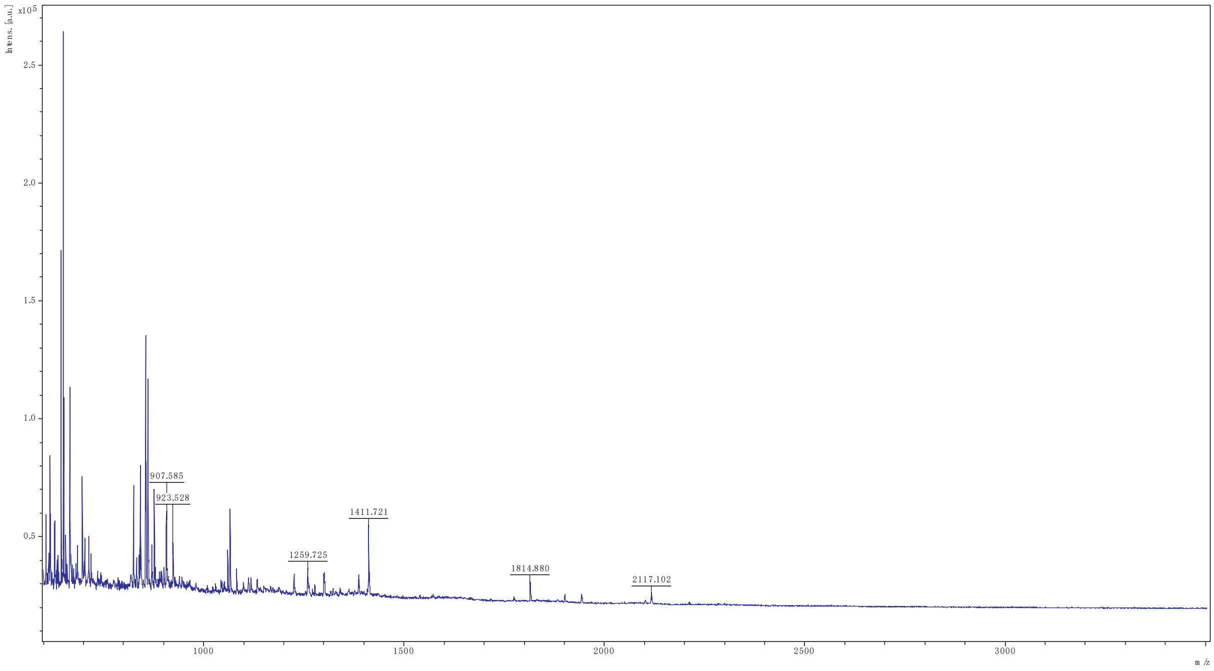

m/z

907.585

923.528

1225.686

1259.725

1386.735

1411.721

1814.880

2117.102

Spot 7 → Legumin J Like

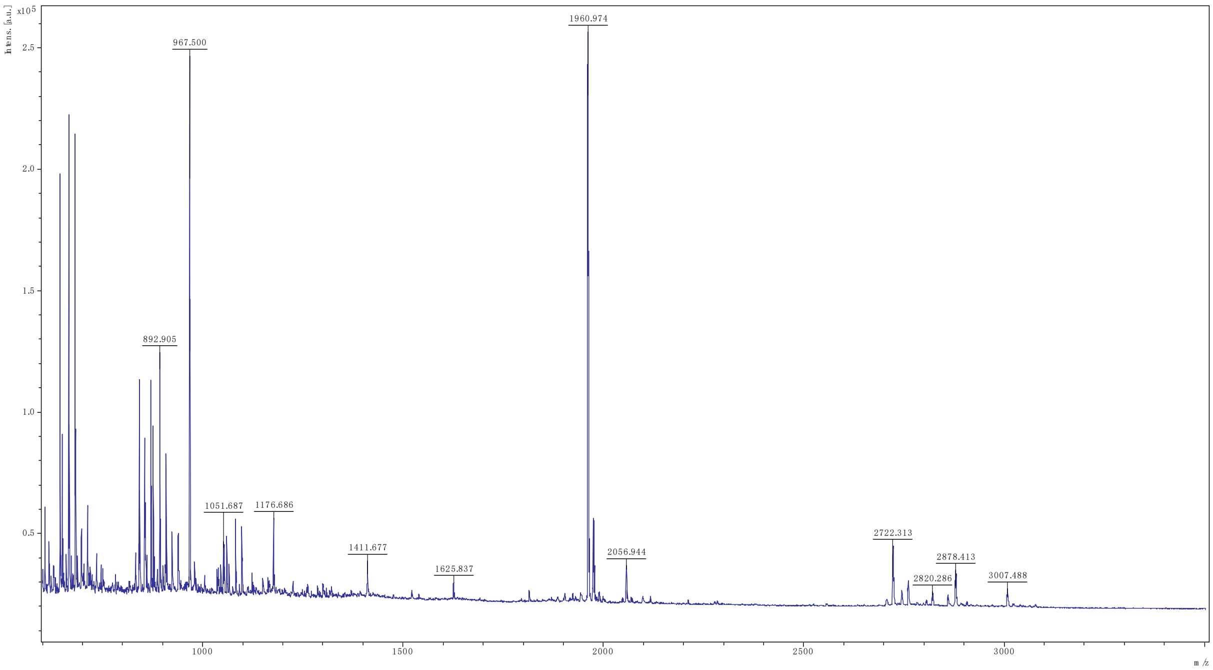

m/z

892.905

908.878

923.404

939.336

967.500

1051.687

1053.486

1081.918

1097.879

1176.686

1411.677

1625.837

1960.721

1960.974

1974.986

1977.984

1988.974

2056.676

2056.944

2720.958

2722.313

2744.189

2759.940

2820.286

2877.015

2878.413

3007.488

Spot 7 → Legumin J like

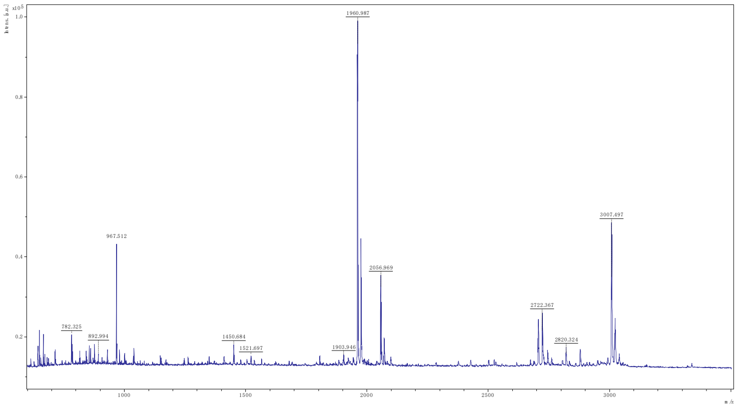

m/z

782.325

892.994

930.455

967.512

979.510

1000.457

1450.684

1521.697

1903.946

1960.987

1975.000

2056.969

2068.921

2070.974

2705.482

2722.367

2744.322

2820.324

2877.431

3007.497

3021.498

3038.480
